# Methyl 3,5-dibromo-4-methyl­benzoate

**DOI:** 10.1107/S1600536810011062

**Published:** 2010-03-31

**Authors:** Aamer Saeed, Hummera Rafique, Jim Simpson, Zaman Ashraf

**Affiliations:** a Department of Chemistry, Quaid-i-Azam University, Islamabad 45320, Pakistan; b Department of Chemistry, University of Otago, PO Box 56, Dunedin, New Zealand; c Riphah Institute of Pharmaceutical Sciences, Islamabad, Pakistan

## Abstract

In the title compound, C_9_H_8_Br_2_O_2_, the mol­ecule is essentially planar with an r.m.s. deviation of 0.0652 Å from the mean plane through all non-H atoms and a dihedral angle of 7.1 (2)° between the benzene ring plane and the carboxyl­ate substituent. In the crystal structure, weak C—H⋯Br hydrogen bonds and weak inter­molecular O⋯Br contacts [3.095 (2) Å], link adjacent mol­ecules into layers parallel to (102). Additional weak inter­molecular C—H⋯O hydrogen bond inter­actions stack the layers above and below the mol­ecular plane and down the *a* axis.

## Related literature

For use of the title compound in the synthesis of natural products, see: Gray & Whalley (1971 ▶); Saeed & Rama (1994 ▶); Harris & Mantle (2001 ▶); Simpson (1978 ▶). For related structures, see: Moorthy *et al.* (2002 ▶); Fan *et al.* (2005 ▶). For inter­molecular O⋯Br contacts, see: Choi *et al.* (2010*a* ▶,*b* ▶); Politzer *et al.* (2007 ▶). For bond-length data, see: Allen *et al.* (1987 ▶).
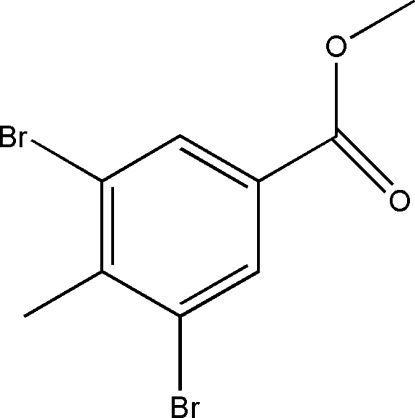


## Experimental

### 

#### Crystal data

C_9_H_8_Br_2_O_2_
*M* *_r_* = 307.97
Orthorhombic, 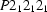

*a* = 3.9716 (2) Å
*b* = 14.2359 (7) Å
*c* = 17.2893 (8) Å
*V* = 977.52 (8) Å^3^
*Z* = 4
Mo *K*α radiation
μ = 8.26 mm^−1^
*T* = 89 K
0.64 × 0.14 × 0.08 mm

#### Data collection

Bruker APEXII CCD diffractometer
Absorption correction: multi-scan (*SADABS*; Bruker, 2006 ▶) *T*_min_ = 0.295, *T*_max_ = 1.000
17658 measured reflections
3471 independent reflections
2922 reflections with *I* > 2σ(*I*)
*R*_int_ = 0.061

#### Refinement

*R*[*F* ^2^ > 2σ(*F* ^2^)] = 0.033
*wR*(*F* ^2^) = 0.065
*S* = 1.09
3471 reflections
120 parameters
H-atom parameters constrained
Δρ_max_ = 1.15 e Å^−3^
Δρ_min_ = −1.09 e Å^−3^
Absolute structure: Flack (1983 ▶), 1659 Friedel pairs
Flack parameter: 0.039 (14)

### 

Data collection: *APEX2* (Bruker, 2006 ▶); cell refinement: *APEX2* and *SAINT* (Bruker, 2006 ▶); data reduction: *SAINT*; program(s) used to solve structure: *SHELXS97* (Sheldrick, 2008 ▶); program(s) used to refine structure: *SHELXL97* (Sheldrick, 2008 ▶) and *TITAN2000* (Hunter & Simpson, 1999 ▶); molecular graphics: *SHELXTL* (Sheldrick, 2008 ▶) and *Mercury* (Macrae *et al.*, 2008 ▶); software used to prepare material for publication: *SHELXL97*, *enCIFer* (Allen *et al.*, 2004 ▶), *PLATON* (Spek, 2009 ▶) and *publCIF* (Westrip, 2010 ▶).

## Supplementary Material

Crystal structure: contains datablocks global, I. DOI: 10.1107/S1600536810011062/jj2027sup1.cif

Structure factors: contains datablocks I. DOI: 10.1107/S1600536810011062/jj2027Isup2.hkl

Additional supplementary materials:  crystallographic information; 3D view; checkCIF report

## Figures and Tables

**Table 1:** Hydrogen-bond geometry (Å, °)

*D*—H⋯*A*	*D*—H	H⋯*A*	*D*⋯*A*	*D*—H⋯*A*
C8—H8*C*⋯O2^i^	0.98	2.70	3.546 (5)	145
C6—H6⋯Br2^ii^	0.95	2.93	3.838 (3)	159
C8—H8*A*⋯O1^iii^	0.98	2.69	3.647 (4)	167

Symmetry codes: (i) 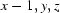; (ii) 
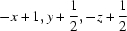
; (iii) 
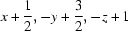
.

**Fig. 1.**
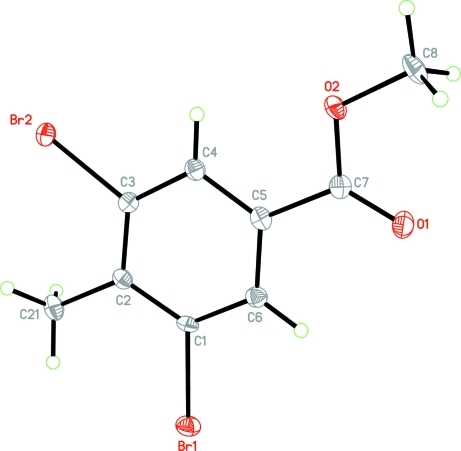
The structure of (I) with displacement ellipsoids for the non-hydrogen atoms drawn at the 50% probability level.

**Fig. 2.**
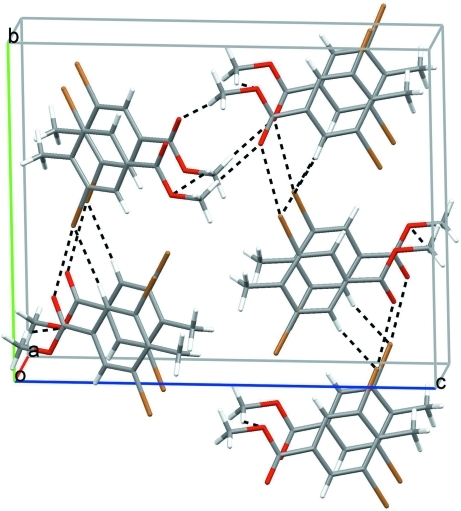
Crystal packing for (I) viewed down the a axis with weak hydrogen bonds and weak O···Br contacts drawn as dashed lines.

## supplementary crystallographic information

### Comment

The title ester, (I), Fig. 1, is an important intermediate towards synthesis of 3,5-dimethoxyphenylacetic acid, a key intermediate in the synthesis of a variety of natural products. These include the sclerotiorin group of fungal metabolites (Gray & Whalley, 1971), isochromans related to sclerotiorin pigments (Saeed & Rama, 1994) and isocoumarins like 7-methylmellein (Harris & Mantle, 2001) and stellatin (Simpson, 1978). C_9_H_8_O_2_Br_2_, (I), was prepared by bromination of methyl 4-methylbenzoate in presence of anhydrous aluminum chloride using an excess of calalyst and no solvent.

The molecule is essentially flat with an rms deviation of 0.0652 Å from the mean plane through all non-hydrogen atoms. The dihedral angle between the C1···C6 ring plane and that of the C7/O1/O2/C8 carboxylate unit is 7.1 (2)°. Bond distances in the molecule are normal (Allen *et al.*, 1987) and comparable to those in related structures (Moorthy *et al.*, 2002; Fan *et al.*, 2005).

In the crystal structure weak intermolecular C6—H6···Br2 hydrogen bonds and weak O1···Br2 contacts at 3.095 (2)Å (Choi *et al.*, 2010a,b; Politzer *et al.*, 2007) link adjacent molecules into layers parallel to the (102) plane. Additional weak intermolecular C8–H8A···O1 and C8–H8C···O2 hydrogen bond interactions involving the carboxylate methyl group stack these layers above and below the molecular plane and down the *a* axis, Table 1, Fig. 2.

### Experimental

Anhydrous aluminum chloride (1.60 mmol) was added portionwise to stirred methyl 4-methylbenzoate (0.6 mmol) at 0°C under a nitrogen atmosphere. Bromine was added over 45 min. and the mixture was further stirred for 30 min at room temperature and at 80 °C for 1 h. The mixture was cooled to room temperature, treated with cold methanol (100 ml) and then stirred overnight. The crude product was filtered and washed with methanol at 30°C then recrystallized from methanol at 10°C to to afford the title compound (86%) as colourless crystals: Anal. calcd. for C_9_H_8_Br_2_O_2_: C, 35.10; H, 2.62; found: C, 35.23; H, 2.67 %

### Refinement

H-atoms were positioned geometrically and refined using a riding model with d(C—H) = 0.95 Å, U_iso_ = 1.2U_eq_ (C) for aromatic and d(C—H) = 0.98 Å, U_iso_ = 1.5U_eq_ (C) for methyl C atoms.

### Crystal data

**Table:**

C_9_H_8_Br_2_O_2_	*F*(000) = 592
*M**_r_* = 307.97	*D*_x_ = 2.093 Mg m^−^^3^
Orthorhombic, *P*2_1_2_1_2_1_	Mo *K*α radiation, λ = 0.71073 Å
Hall symbol: P 2ac 2ab	Cell parameters from 4848 reflections
*a* = 3.9716 (2) Å	θ = 2.8–30.4°
*b* = 14.2359 (7) Å	µ = 8.26 mm^−^^1^
*c* = 17.2893 (8) Å	*T* = 89 K
*V* = 977.52 (8) Å^3^	Rectangular plate, colourless
*Z* = 4	0.64 × 0.14 × 0.08 mm

### Data collection

**Table:**

Bruker APEXII CCD diffractometer	3471 independent reflections
Radiation source: fine-focus sealed tube	2922 reflections with *I* > 2σ(*I*)
graphite	*R*_int_ = 0.061
ω scans	θ_max_ = 33.3°, θ_min_ = 3.7°
Absorption correction: multi-scan (*SADABS*; Bruker, 2006)	*h* = −6→4
*T*_min_ = 0.295, *T*_max_ = 1.000	*k* = −21→21
17658 measured reflections	*l* = −25→25

### Refinement

**Table:**

Refinement on *F*^2^	Secondary atom site location: difference Fourier map
Least-squares matrix: full	Hydrogen site location: inferred from neighbouring sites
*R*[*F*^2^ > 2σ(*F*^2^)] = 0.033	H-atom parameters constrained
*wR*(*F*^2^) = 0.065	*w* = 1/[σ^2^(*F*_o_^2^) + 0.5972*P*] where *P* = (*F*_o_^2^ + 2*F*_c_^2^)/3
*S* = 1.09	(Δ/σ)_max_ = 0.001
3471 reflections	Δρ_max_ = 1.15 e Å^−^^3^
120 parameters	Δρ_min_ = −1.09 e Å^−^^3^
0 restraints	Absolute structure: Flack (1983), 1659 Friedel pairs
Primary atom site location: structure-invariant direct methods	Flack parameter: 0.039 (14)

### Special details

**Table:**

Geometry. All esds (except the esd in the dihedral angle between two l.s. planes) are estimated using the full covariance matrix. The cell esds are taken into account individually in the estimation of esds in distances, angles and torsion angles; correlations between esds in cell parameters are only used when they are defined by crystal symmetry. An approximate (isotropic) treatment of cell esds is used for estimating esds involving l.s. planes.
Refinement. Refinement of *F*^2^ against ALL reflections. The weighted *R*-factor wR and goodness of fit *S* are based on *F*^2^, conventional *R*-factors *R* are based on *F*, with *F* set to zero for negative *F*^2^. The threshold expression of *F*^2^ > σ(*F*^2^) is used only for calculating *R*-factors(gt) etc. and is not relevant to the choice of reflections for refinement. *R*-factors based on *F*^2^ are statistically about twice as large as those based on *F*, and *R*- factors based on ALL data will be even larger.

### Fractional atomic coordinates and isotropic or equivalent isotropic displacement parameters (Å^2^)

**Table:**

	*x*	*y*	*z*	*U*_iso_*/*U*_eq_	
Br1	0.86943 (10)	0.87630 (2)	0.134557 (17)	0.01774 (7)	
C1	0.7685 (7)	0.7603 (2)	0.18453 (18)	0.0116 (6)	
C2	0.8609 (8)	0.67455 (19)	0.15060 (16)	0.0119 (5)	
C21	1.0349 (8)	0.6680 (2)	0.07366 (19)	0.0172 (7)	
H21A	1.2073	0.6188	0.0757	0.026*	
H21B	1.1411	0.7283	0.0615	0.026*	
H21C	0.8697	0.6525	0.0335	0.026*	
C3	0.7704 (8)	0.5947 (2)	0.19236 (18)	0.0120 (6)	
Br2	0.86513 (9)	0.47350 (2)	0.151431 (17)	0.01548 (7)	
C4	0.6050 (9)	0.59686 (19)	0.26311 (17)	0.0128 (5)	
H4	0.5508	0.5404	0.2897	0.015*	
C5	0.5202 (7)	0.6844 (2)	0.29418 (19)	0.0122 (6)	
C6	0.6011 (9)	0.7667 (2)	0.25473 (17)	0.0138 (6)	
H6	0.5426	0.8262	0.2756	0.017*	
C7	0.3351 (8)	0.69264 (19)	0.36902 (17)	0.0133 (5)	
O1	0.2258 (6)	0.76517 (15)	0.39530 (14)	0.0181 (5)	
O2	0.3030 (6)	0.60888 (15)	0.40440 (12)	0.0163 (5)	
C8	0.1345 (11)	0.6119 (2)	0.47902 (17)	0.0214 (6)	
H8A	0.2618	0.6524	0.5144	0.032*	
H8B	0.1215	0.5483	0.5005	0.032*	
H8C	−0.0933	0.6371	0.4725	0.032*	

### Atomic displacement parameters (Å^2^)

**Table:**

	*U*^11^	*U*^22^	*U*^33^	*U*^12^	*U*^13^	*U*^23^
Br1	0.02028 (15)	0.01578 (13)	0.01716 (15)	−0.00211 (15)	0.00154 (16)	0.00416 (11)
C1	0.0112 (13)	0.0118 (13)	0.0118 (14)	−0.0026 (10)	−0.0006 (11)	0.0039 (11)
C2	0.0084 (10)	0.0148 (11)	0.0126 (13)	−0.0006 (13)	−0.0016 (14)	0.0007 (10)
C21	0.0153 (14)	0.0231 (16)	0.0131 (16)	0.0021 (13)	0.0022 (12)	0.0006 (12)
C3	0.0122 (14)	0.0129 (13)	0.0109 (14)	−0.0004 (11)	−0.0019 (11)	−0.0009 (10)
Br2	0.01713 (14)	0.01478 (12)	0.01454 (14)	0.00131 (14)	0.00229 (15)	−0.00278 (10)
C4	0.0103 (13)	0.0151 (12)	0.0131 (13)	−0.0003 (13)	−0.0004 (13)	−0.0003 (10)
C5	0.0090 (12)	0.0176 (14)	0.0099 (14)	−0.0004 (11)	−0.0032 (11)	−0.0003 (12)
C6	0.0122 (14)	0.0139 (13)	0.0152 (13)	−0.0009 (13)	−0.0006 (13)	0.0002 (10)
C7	0.0139 (14)	0.0161 (13)	0.0099 (12)	−0.0001 (11)	−0.0028 (12)	−0.0013 (10)
O1	0.0206 (12)	0.0177 (11)	0.0160 (11)	0.0043 (9)	0.0027 (9)	−0.0009 (9)
O2	0.0208 (13)	0.0181 (10)	0.0098 (10)	−0.0014 (9)	0.0048 (9)	0.0003 (8)
C8	0.0235 (16)	0.0295 (16)	0.0113 (14)	−0.0020 (18)	0.0063 (16)	0.0020 (12)

### Geometric parameters (Å, °)

**Table:**

Br1—C1	1.907 (3)	C4—H4	0.9500
C1—C6	1.387 (4)	C5—C6	1.393 (4)
C1—C2	1.403 (4)	C5—C7	1.493 (4)
C2—C3	1.394 (4)	C6—H6	0.9500
C2—C21	1.502 (4)	C7—O1	1.209 (3)
C21—H21A	0.9800	C7—O2	1.346 (3)
C21—H21B	0.9800	O1—Br2^i^	3.095 (2)
C21—H21C	0.9800	O2—C8	1.454 (4)
C3—C4	1.389 (4)	C8—H8A	0.9800
C3—Br2	1.902 (3)	C8—H8B	0.9800
C4—C5	1.398 (4)	C8—H8C	0.9800
			
C6—C1—C2	123.3 (3)	C6—C5—C4	120.4 (3)
C6—C1—Br1	116.1 (2)	C6—C5—C7	118.2 (3)
C2—C1—Br1	120.6 (2)	C4—C5—C7	121.4 (3)
C3—C2—C1	115.2 (3)	C1—C6—C5	119.0 (3)
C3—C2—C21	121.8 (3)	C1—C6—H6	120.5
C1—C2—C21	123.0 (3)	C5—C6—H6	120.5
C2—C21—H21A	109.5	O1—C7—O2	123.5 (3)
C2—C21—H21B	109.5	O1—C7—C5	124.7 (3)
H21A—C21—H21B	109.5	O2—C7—C5	111.8 (2)
C2—C21—H21C	109.5	C7—O1—Br2^i^	139.7 (2)
H21A—C21—H21C	109.5	C7—O2—C8	114.9 (2)
H21B—C21—H21C	109.5	O2—C8—H8A	109.5
C4—C3—C2	124.0 (3)	O2—C8—H8B	109.5
C4—C3—Br2	116.2 (2)	H8A—C8—H8B	109.5
C2—C3—Br2	119.7 (2)	O2—C8—H8C	109.5
C3—C4—C5	118.2 (3)	H8A—C8—H8C	109.5
C3—C4—H4	120.9	H8B—C8—H8C	109.5
C5—C4—H4	120.9		
			
C6—C1—C2—C3	0.4 (4)	C2—C1—C6—C5	0.2 (5)
Br1—C1—C2—C3	−179.4 (2)	Br1—C1—C6—C5	−180.0 (2)
C6—C1—C2—C21	178.5 (3)	C4—C5—C6—C1	−0.4 (4)
Br1—C1—C2—C21	−1.3 (4)	C7—C5—C6—C1	−179.0 (3)
C1—C2—C3—C4	−0.9 (5)	C6—C5—C7—O1	6.2 (5)
C21—C2—C3—C4	−179.0 (3)	C4—C5—C7—O1	−172.5 (3)
C1—C2—C3—Br2	177.7 (2)	C6—C5—C7—O2	−173.9 (3)
C21—C2—C3—Br2	−0.4 (4)	C4—C5—C7—O2	7.5 (4)
C2—C3—C4—C5	0.7 (5)	O2—C7—O1—Br2^i^	−172.96 (18)
Br2—C3—C4—C5	−177.9 (2)	C5—C7—O1—Br2^i^	7.0 (5)
C3—C4—C5—C6	−0.1 (4)	O1—C7—O2—C8	−2.0 (4)
C3—C4—C5—C7	178.6 (3)	C5—C7—O2—C8	178.1 (3)

Symmetry codes: (i) −*x*+1, *y*+1/2, −*z*+1/2.

### Hydrogen-bond geometry (Å, °)

**Table:**

*D*—H···*A*	*D*—H	H···*A*	*D*···*A*	*D*—H···*A*
C8—H8C···O2^ii^	0.98	2.70	3.546 (5)	145
C6—H6···Br2^i^	0.95	2.93	3.838 (3)	159
C8—H8A···O1^iii^	0.98	2.69	3.647 (4)	167

Symmetry codes: (ii) *x*−1, *y*, *z*; (i) −*x*+1, *y*+1/2, −*z*+1/2; (iii) *x*+1/2, −*y*+3/2, −*z*+1.
